# Foodborne Illness Outbreaks Reported to National Surveillance, United States, 2009–2018

**DOI:** 10.3201/eid2806.211555

**Published:** 2022-06

**Authors:** Alice E. White, Alexandra R. Tillman, Craig Hedberg, Beau B. Bruce, Michael Batz, Scott A. Seys, Daniel Dewey-Mattia, Michael C. Bazaco, Elaine Scallan Walter

**Affiliations:** Colorado School of Public Health, Aurora, Colorado, USA (A.E. White, A.R. Tillman, E. Scallan Walter);; University of Minnesota, Minneapolis, Minnesota, USA (C. Hedberg);; Centers for Disease Control and Prevention, Atlanta, Georgia, USA (B.B. Bruce, D. Dewey-Mattia);; US Food and Drug Administration, College Park, Maryland, USA (M. Batz, M.C. Bazaco);; US Department of Agriculture Food Safety Inspection Service, Washington, DC, USA (S.A. Seys)

**Keywords:** food safety, foodborne diseases, infectious disease outbreaks, enteric infections, public health surveillance, Shiga toxin–producing *Escherichia coli*, *Salmonella*, norovirus, bacteria, United States, viruses

## Abstract

Foodborne outbreaks reported to national surveillance systems represent a subset of all outbreaks in the United States; not all outbreaks are detected, investigated, and reported. We described the structural factors and outbreak characteristics of outbreaks reported during 2009–2018. We categorized states (plus DC) as high (highest quintile), middle (middle 3 quintiles), or low (lowest quintile) reporters on the basis of the number of reported outbreaks per 10 million population. Analysis revealed considerable variation across states in the number and types of foodborne outbreaks reported. High-reporting states reported 4 times more outbreaks than low reporters. Low reporters were more likely than high reporters to report larger outbreaks and less likely to implicate a setting or food vehicle; however, we did not observe a significant difference in the types of food vehicles identified. Per capita funding was strongly associated with increased reporting. Investments in public health programming have a measurable effect on outbreak reporting.

Foodborne diseases remain a major public health challenge in the United States, where 31 known pathogens cause an estimated 9 million illnesses, 56,000 hospitalizations, and 1,300 deaths annually (*1*). Efforts to improve food safety and reduce the burden of foodborne disease rely on data from foodborne disease surveillance and outbreak investigations to help prioritize food safety interventions, policies, and practices. Data from foodborne illness outbreaks reported to the Centers for Disease Control and Prevention (CDC) provide vital information on the foods causing illness and common food–pathogen pairs. Those data are used by the Interagency Food Safety Analytics Collaboration (IFSAC) to inform outbreak-based attribution models that attribute illnesses to specific food categories (*2*,*3*).

Foodborne illness outbreaks are investigated by local, state, and territorial health departments, CDC, the US Food and Drug Administration (FDA), and the Food Safety and Inspection Service of the United States Department of Agriculture and are reported to CDC’s Foodborne Disease Outbreak Reporting Surveillance System (FDOSS) through the web-based National Outbreak Reporting System (NORS). Although reported outbreaks are a rich data source, they represent a subset of all outbreaks occurring in the United States; not all outbreaks will be detected, investigated, and reported. Factors influencing which outbreaks are detected, investigated, and reported to CDC include both structural factors associated with the jurisdiction in which the outbreak occurred (e.g., infrastructure and capacity) and characteristics of the outbreak (e.g., size, geographic location, pathogen).

We integrated data from a variety of sources to examine structural factors and describe outbreak characteristics of foodborne outbreaks involving *Salmonella*, Shiga toxin–producing *Escherichia coli* (STEC) O157, norovirus, and bacterial toxins that were reported to national surveillance. In addition, we assessed the effects of state variation in outbreak reporting on the types of food vehicles identified.

## Methods

### Foodborne Outbreak Data

We obtained outbreak surveillance data from CDC’s FDOSS for 2009–2018, extracted November 22, 2019. This passive surveillance system receives outbreak reports from state, local, and territorial health agencies using a standard outbreak report form that includes information on the date and location of the outbreak, investigation methods, case demographics, etiology, transmission route, setting, and implicated food, among other variables. Forms have been submitted electronically through NORS since 2009. For this study, we included all single-state foodborne outbreaks (exposures occurred in 1 state) reported to FDOSS by 50 states and Washington, DC. We excluded multistate outbreaks (exposures occurred in multiple states) because there are relatively few multistate outbreaks, and single-state outbreaks are more reflective of individual state resources and capacity. We included city jurisdictions reporting independently in state totals. When categorizing outbreaks by pathogen, we included any outbreaks with a confirmed or suspected etiology of *Salmonella*, STEC O157, norovirus, and bacterial toxins (*Clostridium perfringens*, *Bacillus cereus*, and *Staphylococcus aureus*). Outbreaks associated with other priority IFSAC pathogens, including *Campylobacter* spp. and *Listeria monocytogenes* were not included in pathogen-specific analyses because few outbreaks were reported. We included outbreaks caused by multiple pathogens in all outbreaks and excluded them from pathogen group analysis.

We calculated outbreak reporting rates as the number of single-state foodborne illness outbreaks reported annually per 10 million population for 2009–2018, averaged over time by state. We categorized states by outbreak reporting quintile for all etiologies, then collapsed into high (the highest outbreak reporting quintile), middle (the middle 3 quintiles), or low (the lowest quintile) reporting groups. We compared high, middle, and low outbreak reporting over time and by the structural and outbreak characteristics described using bivariate χ^2^, Kruskal-Wallis, or Fisher exact test as appropriate. We analyzed data using SAS version 9.4 (SAS Institute Inc., https://www.sas.com). This analysis did not meet the definition of human subjects research as defined in the US Code of Federal Regulations, Title 45 Part 46, and was not subject to review by an institutional review board.

### Structural Characteristics

Structural characteristics related to state reporters were available from a variety of sources and included reporting structure, funding sources, and participation in foodborne or environmental health programs. Reporters were classified as having a centralized surveillance reporting structure, in which state health departments were the primary leaders of surveillance and outbreak investigations, or decentralized structure, in which local health departments were the primary leaders of surveillance and outbreak investigations using the 2014 LawAtlas codebook for state foodborne illness reporting laws and the 2007 Enteric Disease Outbreak Investigation and Surveillance survey (*4*,*5*). Funding sources we examined included the total number of public health full-time employees (FTEs) and state public health revenue by source, available from the Association of State and Territorial Health Officials (ASTHO) Profile of State and Territorial Public Health reports (https://www.astho.org); Epidemiology and Laboratory Capacity for Prevention and Control of Emerging Infectious Diseases (ELC) cooperative-agreement funding for fiscal years 2016–2018, which funded states and territories to detect, respond to, control, and prevent infectious diseases (*6*); and federal foodborne or environmental health programs. ELC-funded state programs for foodborne illness detection and response include the Integrated Food Safety Centers of Excellence (CoE; https://www.cdc.gov/foodsafety/centers), Foodborne Diseases Centers for Outbreak Response Enhancement (FoodCORE; https://www.cdc.gov/foodcore), and OutbreakNet Enhanced (OBNE, https://www.cdc.gov/foodsafety/outbreaknetenhanced). States can receive funding for multiple programs. For analysis purposes, we assigned states to the program with the highest average funding award per capita (e.g., states with CoE and FoodCORE or OBNE were categorized as CoE). ELC-funded state programs for norovirus included Norovirus Sentinel Testing and Tracking (NoroSTAT, https://www.cdc.gov/norovirus/reporting/norostat). State programs for foodborne illness funded by CDC under the Emerging Infections Program included the Foodborne Diseases Active Surveillance Network (FoodNet; https://www.cdc.gov/foodnet). Environmental health outbreak response programs included FDA Voluntary National Retail Regulatory Food Program Standard 5, state-level meat and poultry inspection, FDA Rapid Response Team, Environmental Health Specialists Network, and the National Environmental Assessment Reporting System; the CIFOR Food Safety Programs Reference Guide contains program descriptions (*7*). We obtained surveillance data for state estimates of *Salmonella* and STEC O157 illnesses from the Laboratory-based Enteric Disease Surveillance (LEDS) system (*8*,*9*) and used them to compare underlying disease rates with outbreak reporting.

### Outbreak Characteristics

We obtained outbreak characteristics from FDOSS. Characteristics included the number of ill cases per outbreak (laboratory-confirmed and probable primary cases); setting identified (yes/no); setting type (restaurant, private residence, institution, or other); food implicated (yes/no); food implicated using food categories defined by IFSAC (*10*); and whether the implicated food was confirmed or suspected. During 2017 and 2018, states reported foods as confirmed or suspected directly to NORS. For outbreaks before 2017, in this analysis we retrospectively classified implicated foods as confirmed or suspected using criteria outlined in the current NORS guidance (https://www.cdc.gov/nors/forms.html).

## Results

During 2009–2018, a total of 8,131 single-state outbreaks involving 131,525 outbreak-associated illnesses were reported. Of these, 5,986 (74%) had a confirmed or suspected etiology. Causes of the outbreaks included norovirus (2,798; 47%), *Salmonella* (1,191; 20%), bacterial toxins (617; 10%), and STEC O157 (150; 3%) (Table 1). The etiology was confirmed for 49% of the outbreaks (range across states 21%–84%). The percentage of outbreaks with a confirmed etiology was higher for *Salmonella* (92%) and STEC (93%) outbreaks than for norovirus (55%) and bacterial toxin (42%) outbreaks. A confirmed or suspected food vehicle was identified for 36% of the total outbreaks (range by state 11%–77%) (Table 1).

**Table 1:** Single-state foodborne outbreaks reported by US states and Washington, DC, to the Foodborne Disease Outbreak Surveillance System, 2009–2018*

Characteristic	All etiologies	Norovirus	*Salmonella*	Bacterial toxins	STEC O157
No. reporters	51	51	50	46	34
No. outbreaks	8,131	2,798	1,191	617	150
Range by state	9–906	1–357	1–100	1–72	1–14
Total outbreak-associated illnesses	131,525	55,406	21,656	17,110	1,624
Range by state	84–11,242	22–4,755	3–1,717	5–1,771	2–164
Mean annual outbreak rate per 10 million population, by state	28.6	9.2	4.7	2.6	0.9
Range by state	4.7–86.3	0.5–52.1	1.3–11.4	0.1–7.6	0.1–3.2
Outbreaks with confirmed etiology, no. (%)	3,962 (49)	1,529 (55)	1,101 (92)	258 (42)	139 (93)
Range by state, %	21–84	0–100	54–100	0–100	50–100
Outbreaks with food vehicle identified, no. (%)	2,960 (36)	693 (25)	477 (40)	397 (64)	88 (59)
Range by state, %	11–77	0–100	0–80	0–100	0–100
Outbreaks with confirmed etiology and food vehicle identified, no. (%)	1,819 (22)	425 (15)	449 (38)	194 (31)	82 (55)
Range by state, %	0–56	0–40	0–80	0–100	0–80

*All etiologies includes reported outbreaks with multiple etiologies. Bacterial toxins include *Clostridium perfringens*,* Bacillus cereus*, and *Staphylococcus aureus. *STEC, Shiga toxin–producing *Escherichia coli.*

**Figure 1:**
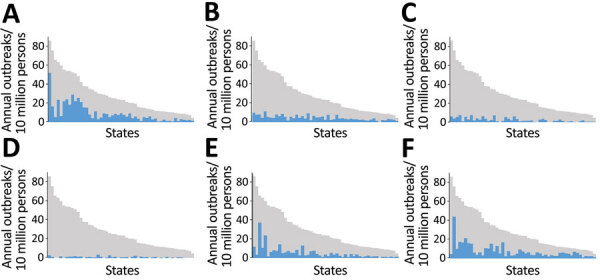
Mean annual rates of foodborne disease outbreaks reported to the Centers for Disease Control and Prevention per 10 million population by etiology and US state (deidentified), Foodborne Disease Outbreak Surveillance System, United States, 2009–2018. Blue bars represent outbreaks reported for the specified etiology. Gray bars represent all outbreaks reported. Blue and gray bars correspond to the same reporting jurisdiction and are ordered by reporting rate for all single-state outbreaks. A) Norovirus; B) *Salmonella*; C) bacterial toxins; D) Shiga toxin–producing *E. coli* O157; E) Other known cause; F) Unknown cause.

**Figure 2:**
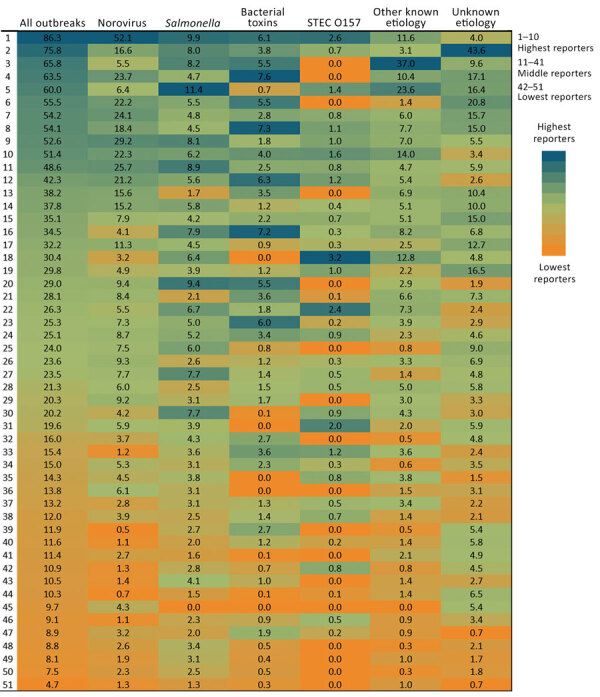
Annual rates of foodborne-illness outbreaks per 10 million population by reporting state and etiology, Foodborne Disease Outbreak Surveillance System, United States, 2009–2018. STEC, Shiga toxin–producing *Escherichia coli.*

Overall, states reported a mean of 29 outbreaks per 10 million population per year (range by state: 5–86 outbreaks) and a mean of 9 (range 0.5–52) norovirus outbreaks, 5 *Salmonella* (range 1–11) outbreaks, 3 (range 0.1–8) bacterial toxin outbreaks, and 0.9 (range 0.1–3) STEC O157 outbreaks per 10 million population per year (Table 1; Figure 1). The 10 states with the highest number of reported outbreaks (high reporters) averaged 62 outbreaks per 10 million population per year, whereas the 10 states with the fewest number of reported outbreaks (low reporters) averaged 9 and the remaining 30 states (middle reporters) 24 outbreaks per 10 million population per year (Figure 2). Outbreak reporting quintiles were mostly consistent across pathogens, with the exception of STEC O157 (Figure 2). Among outbreaks with a known etiology other than norovirus, *Salmonella*, bacterial toxins, and STEC O157, the most common etiologies were fish toxins (433 outbreaks, 33%) and *Campylobacter* (294 outbreaks, 22%).

**Figure 3:**
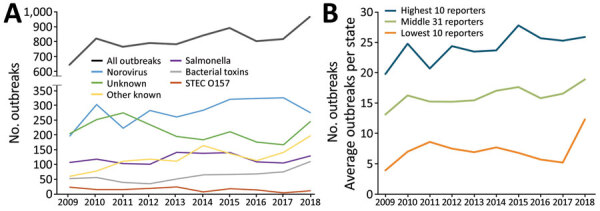
Foodborne outbreaks reported through the Foodborne Disease Outbreak Surveillance System, by etiology and reporting group, United States, 2009–2018. A) Single-state foodborne outbreaks by etiology. B) Average outbreaks per state by reporting group (high, middle, low). STEC, Shiga toxin–producing *Escherichia coli.*

During 2009–2018, low reporters reported less than one third the number of outbreaks (624) reported by high reporters (2,416) (Table 2). This pattern was similar over time except in 2017–2018, when the number of outbreaks reported by low reporters more than doubled as a result of changes in reporting practices in a single large-population state (Figure 3, panels A, B). Low reporters were significantly less likely than middle and high reporters to report outbreaks with an identified etiology (57% low, 73% middle, 79% high) and reported fewer norovirus outbreaks (5% low, 60% middle, 36% high). Low reporters were also less likely to identify a setting (73% low, 92% middle, 96% high) and less likely to implicate (26% low, 38% middle, 36% high) or confirm (56% low, 75% middle, 75% high) a food vehicle. Low reporters were more likely to report the sex of case-patients as unknown (low 13%, 9% middle, 8% high). Low reporters were also more likely to report larger outbreaks (median for low, 10 cases; middle, 8 cases; high, 6 cases) (Table 2). These trends were similar across all pathogen groups.

**Table 2:** Outbreak characteristics from high, middle, and low outbreak reporter states, all etiologies, Foodborne Disease Outbreak Surveillance System, United States, 2009–2018*

Characteristic	Highest 10 reporters	Middle 31 reporters	Lowest 10 reporters	p value
Total no. outbreaks	2,416	5,091	624	
Etiology identified	1,897 (78.5)	3,733 (73.3)	356 (57.1)	<0.01
Confirmed etiology‡				<0.01
Norovirus	546 (35.7)	913 (59.7)	70 (4.6)	
Salmonella	245 (22.3)	731 (66.4)	125 (11.4)	
Bacterial toxins†	67 (26.0)	167 (64.7)	24 (9.3)	
STEC O157	44 (31.7)	87 (62.6)	8 (5.8)	
Other known§	257 (27.5)	642 (68.7)	36 (3.9)	
Confirmed or suspected				<0.01
Norovirus	1,036 (37.0)	1,661 (59.4)	101 (3.6)	
Salmonella	264 (22.2)	782 (65.7)	145 (12.2)	
Bacterial toxins†	168 (27.2)	416 (67.4)	33 (5.3)	
STEC O157	48 (32.0)	92 (61.3)	10 (6.7)	
Other known§	381 (31.0)	782 (63.6)	67 (5.4)	
Setting identified	2,310 (95.6)	4,678 (91.9)	457 (73.2)	<0.01
Setting‡¶				<0.01
Restaurant	1,528 (66.2)	2,893 (61.8)	237 (51.9)	
Institution	78 (3.4)	186 (4.0)	31 (6.8)	
Private residence	217 (9.4)	366 (7.8)	45 (9.9)	
Other single setting	119 (5.2)	303 (6.5)	32 (7.0)	
Multiple setting	368 (15.9)	930 (19.9)	112 (24.5)	
Food vehicle confirmed or suspected	879 (36.4)	1,917 (37.7)	164 (26.3)	<0.01
Food‡				<0.01
Multiple	314 (35.7)	704 (36.7)	73 (44.5)	
Aquatic animals	192 (21.8)	335 (17.5)	11 (6.7)	
Land animals	214 (24.4)	522 (27.2)	51 (31.1)	
Plant	138 (15.7)	290 (15.1)	24 (14.6)	
Other#	21 (2.4)	66 (3.4)	5 (3.1)	
Food vehicle confirmed	656 (74.6)	1,440 (75.1)	92 (56.1)	<0.01
Season				0.02
Winter	649 (26.9)	1,306 (25.7)	128 (20.5)	
Spring	639 (26.5)	1,481 (29.1)	195 (31.3)	
Summer	613 (25.4)	1,282 (25.2)	166 (26.6)	
Autumn	515 (21.3)	1,022 (20.1)	135 (21.6)	
Sex of case-patients unknown	196 (8.1)	443 (8.7)	79 (12.7)	<0.01
No. cases, median (IQR)**	6 (11)	8 (13)	10 (20)	<0.01††

*Values are no. (%) except as indicated. The highest reporter states were the highest outbreak reporting quintile, middle reporters the middle 3 quintiles, and low reporters the lowest quintile, based on number of outbreaks reported per 10 million population. p values are from χ^2^ test results compared across the 3 reporting tiers. STEC, Shiga toxin–producing *Escherichia coli*. †Bacterial toxin outbreaks include *Clostridium perfringens*,* Bacillus cereus*,* Staphylococcus aureus.*‡Among outbreaks with characteristic identified. §Includes outbreaks associated with multiple pathogens. ¶Restaurant setting includes caterer, banquet hall; Institution includes daycares, hospitals, long-term care facilities/nursing homes/assisted living facilities, prison/jails, and school/college/universities; Other setting category includes camp, fair, festival, other temp or mobile services, farm/dairy, grocery store, hotel/motel, office/indoor workplace, other, religious facility, ship/boat. #Includes foods that were unclassifiable or invalid using food categories defined by the Interagency Food Safety Analytics Collaboration (*8*). **Laboratory-confirmed and probable primary cases. ††By Kruskal-Wallis test.

We found no apparent associations between reporting structure and reporting group (Table 3). The percentage of state agency finance received from federal sources was similar across reporting groups, and although high reporters (27%) were more likely than middle reporters (21%) and low reporters (15%) to have received federal funding from CDC, the difference was not statistically significant. Per capita ELC funding was, however, significantly associated with reporting group; high reporters received more funding ($1.30 per capita) than middle reporters ($0.81) and low reporters ($0.44) (p<0.01). Receiving funding for foodborne programs was not statistically significantly associated with reporting group, but all reporters in the highest funding tiers (CoE, FoodCORE) were high or middle reporters, and only 1 of the 15 states receiving no foodborne program funding was a high reporter. Reporters receiving OBNE funding were equally distributed across reporting groups. Similarly, differences between reporters based on whether they receive funding for NoroSTAT or FoodNet were not statistically significant across tiers, but all states receiving funding were high or middle reporters. We saw no association between participation in environmental health outbreak response programs and reporting (Table 3). We observed similar trends for outbreak and structural characteristics across reporters for outbreaks caused by 4 pathogens (Appendix Tables 1–4). The average *Salmonella* incidence rate as reported to LEDS was consistent across reporting groups (Appendix Table 2), whereas high reporters of STEC O157 outbreaks also reported a higher average STEC incidence rate (4.9 illnesses per 100,000 population) compared with middle (2.4) and low (2.6) reporters (p = 0.04) (Appendix Table 4). 

**Table 3:** State structural characteristics from all outbreak reporter states, Foodborne Disease Outbreak Surveillance System, United States, 2009–2018*

Characteristic	Highest 10 reporters	Middle 31 reporters	Lowest 10 reporters	p value
Reporting structure				0.61
No. centralized (%)	5 (50.0)	10 (32.3)	4 (40)	
No. decentralized (%)	5 (50.0)	21 (67.7)	6 (60)	
State agency finance, median (IQR)†				
% State funds	26.9 (22.5–32.6)	29.2 (21.7–47.3)	19.7 (12.9–23.7)	0.11
% Federal funds	54.3 (39.9–63.1)	51.1 (43.8–61.4)	49.4 (39.7–69.0)	0.96
% CDC federal funds	26.7 (23.6–36.5)	20.6 (15–33.3)	14.8 (14.2–28.3)	0.14
Median ELC funding per capita, US$‡	$1.30 ($0.91-2.12)	$0.81 ($0.45–1.49)	$0.44 ($0.34–0.59)	<0.01
State agency workforce				
FTEs per 10,000 population, median (IQR)†	2.7 (2.2–4.9)	2.2 (1.2–5.1)	4.8 (2.6–6.8)	0.37
CDC ELC-funded foodborne programs§				0.35
CoE	3 (30.0)	3 (9.7)	0	
FoodCORE	1 (10.0)	4 (12.9)	0	
OBNE	5 (50.0)	15 (48.4)	5 (50.0)	
None	1 (10.0)	9 (29.3)	5 (50.0)	
NoroSTAT¶	3 (30.0)	9 (29.3)	0	0.18
FoodNet#	3 (30.0)	7 (22.6)	0	0.21
Food safety environmental health programs				
FDA standard 5**	4 (40.0)	15 (48.4)	3 (30.0)	0.63
State-level meat and poultry inspection	4 (40.0)	19 (61.3)	5 (50.0)	0.46
RRT	4 (40.0)	16 (51.6)	4 (40.0)	0.79
EHS-Net	2 (20.0)	3 (9.7)	0	0.36
NEARS	5 (50.0)	15 (48.4)	4 (40.0)	0.93

*Values are no. (%) except as indicated. The highest reporter states were the highest outbreak reporting quintile, middle reporters the middle 3 quintiles, and low reporters the lowest quintile, based on number of outbreaks reported per 10 million population. p values are from Kruskal-Wallis tests for continuous variables and Fisher exact test for categorical variables. CoE, Center of Excellence; ELC, Epidemiology and Laboratory Capacity for Prevention and Control of Emerging Infectious Diseases; FDA, Food and Drug Administration; FTE, full-time employee; NEARS, National Environmental Assessment Reporting System; OBNE, OutbreakNet Enhanced; RRT, FDA Rapid Response Team. †Association of State and Territorial Health Officials Profile of State and Territorial Public Health volume 4. ‡ELC funding per capita, fiscal years 2016–18. Excludes supplemental Zika virus funding for fiscal years 2016–20. §ELC-funded foodborne programs: Integrated Food Safety Centers of Excellence, Foodborne Diseases Centers for Outbreak Response Enhancement (FoodCORE), OBNE;.States with multiple programs were categorized into the category with more funding (e.g., states with CoE and FoodCORE were counted only in CoE) such that program categories are mutually exclusive. ¶Norovirus Sentinel Testing and Tracking (NoroSTAT). #Foodborne Diseases Active Surveillance Network. **State agency participation in FDA Voluntary National Retail Regulatory Food Program Standard 5 (Foodborne Illness and Food Defense Preparedness and Response) as of most recent assessment or audit.

**Figure 4:**
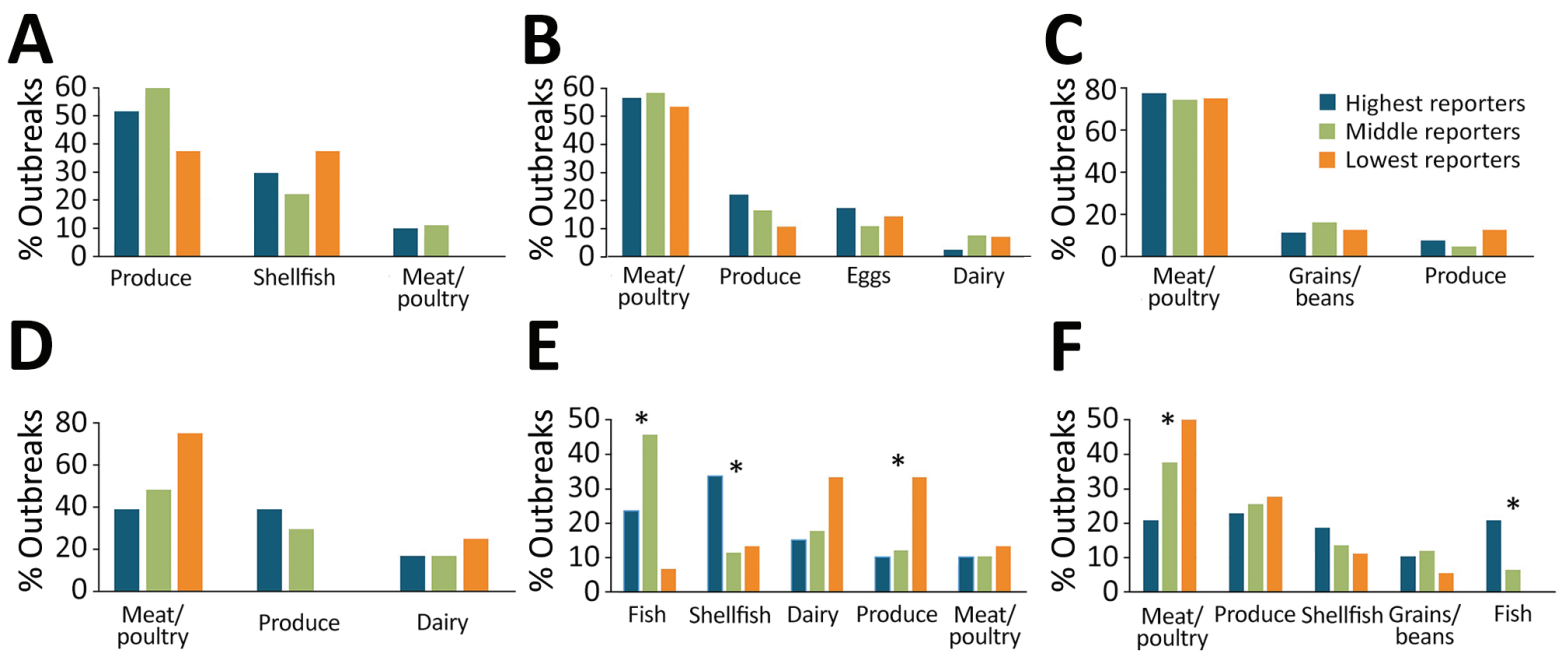
Most common foods implicated in foodborne illness, by detailed food category, Foodborne Disease Outbreak Surveillance System, United States, 2009–2018. Asterisks (*) indicate statistical significance (p<0.05 by Fisher exact test). Data are shown for (A) norovirus, 216 cases; (B) *Salmonella*, 321 cases; (C) bacterial toxins, 209 cases; D) Shiga toxin–producing *Escherichia coli* O157, 76 cases; (E) illness of other known etiology, 715 cases; (F) illness of unknown etiology, 191 cases.

The distribution of implicated foods categorized by Level 1 IFSAC overarching food category (i.e., land animals, aquatic animals, plant), multiple, or other differed substantially by reporting group for all etiologies and other known etiologies, but not for norovirus, *Salmonella*, bacterial toxin, and STEC O157 outbreaks (Table 4). We saw slightly more variation across reporters when implicated foods were classified by more detailed level 2 food type categories (e.g., fish, shellfish, dairy, meat and poultry, eggs, produce, grains and beans); the low reporters reporting fewer produce outbreaks for norovirus, *Salmonella*, and STEC O157 etiology outbreaks, and more meat and poultry outbreaks for STEC O157 and outbreaks of unknown etiology (Figure 4).

**Table 4:** Overarching food categories of implicated food vehicles in outbreaks reported to Foodborne Disease Outbreak Surveillance System, United States, 2009–2018*

Characteristic	Land animals	Aquatic animals	Plants	Unassignable†	Other‡	p value
All etiologies						<0.01
Highest reporters	214 (24.4)	192 (21.8)	138 (15.7)	314 (35.7)	21 (2.4)	
Middle reporters	522 (27.2))	335 (17.5)	290 (15.1)	704 (36.7)	66 (3.4)	
Lowest reporters	51 (31.1)	11 (6.7)	24 (14.6)	73 (44.5)	5 (3.1)	
Norovirus						0.26
Highest reporters	16 (6.5)	27 (10.9)	55 (22.2)	136 (54.8)	14 (5.7)	
Middle reporters	15 (3.5)	31 (7.3)	85 (20.0)	261 (61.4)	33 (7.8)	
Lowest reporters	1 (5.0)	3 (15.0)	4 (20.0)	12 (60.0)	0	
*Salmonella*						0.61
Highest reporters	63 (52.5)	1 (0.8)	18 (15.0)	36 (30.0)	2 (1.7)	
Middle reporters	165 (52.6)	10 (3.2)	41 (13.1)	88 (28.0)	10 (3.2)	
Lowest reporters	22 (51.2)	3 (7.0)	4 (9.3)	12 (27.9)	2 (4.7)	
Bacterial toxins§						0.89
Highest reporters	45 (42.9)	1 (1.0)	10 (9.5)	49 (46.7)	0	
Middle reporters	115 (41.8)	3 (1.1)	31 (11.3)	121 (44.0)	5 (1.8)	
Lowest reporters	6 (35.3)	0	3 (17.7)	8 (47.1)	0	
STEC O157						0.65
Highest reporters	11 (50.0)	0	7 (31.8)	4 (18.2)	0	
Middle reporters	37 (61.7)	1 (1.7)	16 (26.7)	6 (10.0)	0	
Lowest reporters	5 (83.3)	0	0	1 (16.7)	0	
Other known						0.01
Highest reporters	67 (24.5)	143 (52.2)	31 (11.3)	32 (11.7)	1 (0.4)	
Middle reporters	137 (25.6)	265 (49.4)	68 (12.7)	57 (10.6)	9 (1.7)	
Lowest reporters	7 (30.4)	3 (13.0)	7 (30.4)	5 (21.7)	1 (4.4)	
Unknown						0.06
Highest reporters	12 (10.9)	20 (18.2)	17 (15.5)	57 (51.8)	4 (3.6)	
Middle reporters	53 (17.3)	25 (8.1)	49 (16.0)	171 (55.7)	9 (2.9)	
Lowest reporters	10 (18.2)	2 (3.6)	6 (10.9)	35 (63.6)	2 (3.6)	

*Values are no. (%) except as indicated. p values are determined by χ^2^ test. †A food or foods were implicated, but the contaminated ingredient was not determined so a food category could not be assigned or >1 food category was implicated using the Interagency Food Safety Analytics Collaboration categorization scheme (*10*). ‡Includes foods that were unclassifiable using food categories defined by the Interagency Food Safety Analytics Collaboration. §Bacterial toxin outbreaks include *Clostridium perfringens*,* Bacillus cereus*,* Staphylococcus aureus.*

## Discussion

Several factors may affect outbreak reporting. NORS is a passive, voluntary system, and reporting depends on state and local capacity to detect, investigate, and report outbreaks. We found considerable variation across states in the number and types of foodborne outbreaks reported through NORS. The top 10 states reported 4 times more outbreaks per 10 million population than the lowest 10 states reported. The widest gap in outbreak reporting rates was for norovirus outbreaks; the highest reporter reported >40 times as many outbreaks as the lowest one. We also found variation in the types of outbreaks reported by states; low reporters were more likely to report larger outbreaks caused by reportable conditions (e.g., *Salmonella*, STEC O157) and less likely to implicate a setting or food vehicle in an outbreak.

Some differences in outbreak reporting may be due to differences in underlying incidence of disease. For example, most outbreaks associated with fish toxins are inherently regional, occurring in coastal states (*11*), and they tend to be smaller (*12*). In this study, aquatic animal outbreaks were mostly associated with norovirus and were more likely to occur in coastal states. We found that states that reported more STEC O157 outbreaks also reported a higher incidence of STEC to LEDS than middle and low reporters. However, although regional variation is substantial in reported *Salmonella* cases by population overall and among serotypes (*13*), we did not find *Salmonella* outbreak reporting to be correlated with incidence. Despite variation in outbreak reporting across states, we did not identify substantial differences over time or in the foods reported, which suggests that national outbreak surveillance is stable and a reliable source for monitoring relative trends in foodborne illness, including estimating food source attribution.

The structural characteristic most closely associated with outbreak reporting rates was per capita ELC funding. High reporters received ≈3 times as much funding as low reporters. These estimates included funding for nonfoodborne infectious disease program areas, such as healthcare-associated infections and vectorborne disease, suggesting that increasing funding has a positive effect overall on public health department capacity. In foodborne outbreak investigations, epidemiologists work directly on or collaborate with waterborne, animal contact, and other communicable disease programs, especially in local public health agencies. We observed that states that were high reporters were high reporters across multiple pathogen groups, including pathogens detected primarily through reportable disease surveillance (*Salmonella*, STEC O157) and pathogens detected primarily through nonreportable, or complaint-based, surveillance (norovirus, bacterial toxins).

The ability of states to detect outbreaks varies and depends on type of surveillance systems, interview questionnaires, cluster and outbreak tracking systems, case definitions, and laboratory testing capacity. Previous work found a correlation between the number of consumer complaints received by an agency and outbreak reporting rates; however, complaint systems range from no system, to localized systems that do not communicate across jurisdictions, to fully centralized systems (*14*). Jones et al. found that outbreak reporting was higher in states requiring submission of all *Salmonella* isolates to state laboratories and in states that routinely perform molecular subtyping of all isolates (*15*), which has since become standard practice.

Once an outbreak is detected, investigators determine whether they have the resources to proceed with an investigation. Most jurisdictions prioritize investigations associated with pathogens that may cause more severe illness (e.g., STEC O157); however, many lack the personnel to investigate outbreaks of less severe illness or may intentionally deprioritize norovirus outbreaks that are more likely to spread person-to-person, such as in congregate settings (*16*). Furthermore, outbreak investigations are costly (*17*,*18*), requiring time, resources, and commitment among competing priorities (*19*), and some jurisdictions may be less willing to divert personnel and resources from other public health activities or may prioritize outbreaks on the basis of the likelihood of finding actionable information. Cross-disciplinary and interagency collaboration is crucial to successful outbreak investigations; states reporting more outbreaks also reported more collaboration with other states and federal partners (*15*). Finally, states differentially interpret foodborne outbreak and cluster case definitions and report inconsistency and ambiguity in how these definitions are applied for national reporting (*19*).

Although overall ELC funding was associated with increased reporting, we did not find a statistically significant association between reporting and participation in CDC ELC-funded foodborne (CoE, FoodCORE, OBNE) and norovirus (NoroSTAT) programs, CDC foodborne programs funded through other mechanisms (FoodNet), or environmental health programs. This finding could be caused by a delay in observing effects of the funding award. Average funding awards vary within programs, and data on funding for specific foodborne programs were not readily accessible. For example, the average annual award for FoodCORE is $190,000–$510,000 per site, depending on population size and individual work plans (*20*). Funding is awarded through an application process, so awards may reflect capacity and support in the jurisdiction applying, whereas states with less capacity may be less likely to apply for or receive supplemental ELC grant funding. ELC funding awards are competitive and could be an indication of the underlying capacity of public health agencies to conduct surveillance, rather than a specific cause for high reporting of foodborne disease outbreaks.

Outbreak investigations provide critical information on the epidemiology of foodborne diseases and the foods that cause illness. Opportunities to improve outbreak response and reporting are ample, and improvements could further our understanding of what causes foodborne illnesses. Funding is not the only investment needed to improve capacity. Funding must be targeted and flexible (*15*). Peer, community, and cross-jurisdictional support, as fostered by the CoE within the 5 CoE regions (https://www.cdc.gov/foodsafety/centers), are potential mechanisms for improving capacity. Continuing education, workforce engagement, and ongoing evaluation and quality improvement using standardized metrics are all components of increasing public health capacity. Targeted CDC-led funding programs; expansion of CoE-led regional training, mentorship, and technical assistance programs; and opportunities for state- and local-level collaboration via peer networks appear to be useful for improving outbreak surveillance and response. Evaluation using standardized metrics (*7*,*20*) can identify evidence-based practices to continue to make the system more efficient and effective.

Despite variability in reporting, this study found the food categories reported across groups were similar, which supports the use of outbreak data in food source attribution estimates. IFSAC, a collaboration across 3 federal agencies (CDC, FDA, and USDA-FSIS), produces annual estimates of the most common food categories responsible for illnesses caused by pathogens based on national surveillance data for foodborne outbreaks (*2*). However, the extent to which the distribution of food vehicles and locations of preparation implicated in outbreaks reflect the same vehicles and locations as sporadic foodborne illnesses is unknown (*10*). Most foodborne illnesses are not associated with a known outbreak, and the use of outbreak data for attribution may be limited if reported outbreaks are not representative of all foodborne outbreaks (*3*). Our study found that although there is variation in the number and types of outbreaks reported by states as well as an overall low proportion of outbreaks with an implicated food, there was not substantial variation in the foods reported, suggesting the IFSAC approach of using outbreak data for national food source attribution estimates is not biased by state reporting practices. This finding is consistent with other work that found similar characteristics of sporadic and outbreak-associated foodborne illnesses and continues to be an active focus for IFSAC (*21*).

The first limitation of our study is that no data are readily available to identify and describe outbreaks that were detected and investigated but not reported. Some jurisdictions may be more likely to report outbreaks with an identified etiologic agent or food vehicle. Furthermore, in focusing on reporting, this study did not capture other improvements in completeness and timeliness of outbreak response activities. For example, FoodCORE metrics demonstrate improved completeness and timeliness of outbreak investigations (*20*), and this study did not assess the effects of intermediary metrics on national reporting. Limited data were available on state structural characteristics, and our study did not incorporate factors such as laboratory testing metrics, surveillance and investigation practices, and other state or local level established practices. Relevant survey data sources have not been updated in the past decade, including surveys used by Jones, et al., such as the Council of State and Territorial Epidemiologists Food Safety Capacity Assessment and the Association of Public Health Laboratories national PulseNet survey (*22*,*23*), which limited our ability to compare the effects of structural factors over time. Finally, participation in specific foodborne surveillance programs, which we did not find to be significantly associated with reporting, changed over the course of the study period, and our methods did not adjust for changes in participation over time. Specifically, the CoE program was started in 2012, and OBNE was started in 2015.

Future projects should include national surveys that further explore the association between structural factors and detecting, investigating, and reporting foodborne outbreaks. Some data were from different years; for example, ELC funding was only publicly available for 2016–2018. This analysis focused only on reported outbreaks with foodborne transmission, and states likely have different practices for reporting different transmission routes. Finally, this study focused on state-level outbreak reporting. However, most outbreak investigations occur at local public health agencies. Expertise, interest, and preparedness vary dramatically within states, particularly decentralized ones, to respond to foodborne outbreaks. Results from this study did not indicate a relationship between state legal structure and reporting, but this variable does not capture the nuance and diversity of the responsibility of investigating foodborne outbreaks. However, this finding could affect how federal funders such as CDC can target funding to improve communicable disease surveillance and public health preparedness.

In conclusion, this study demonstrates that investments in public health programming produce large benefits and measurable impact on national surveillance. Other studies have shown that robust surveillance systems improve health and decrease overall healthcare costs (*24*). Because individual state characteristics do not appear to bias our detection of which foods are associated with outbreaks, improving outbreak surveillance will also improve food attribution efforts.

Appendix Additional information about foodborne illness outbreaks reported to national surveillance, United States, 2009–2018.

## References

[1] Scallan E, Hoekstra RM, Angulo FJ, Tauxe RV, Widdowson MA, Roy SL, et al. Foodborne illness acquired in the United States—major pathogens. Emerg Infect Dis. 2011;17:7–15. 10.3201/eid1701.P11101 21192848 PMC3375761

[2] Interagency Food Safety Analytics Collaboration. Foodborne illness source attribution estimates for *Salmonella, Escherichia coli* O157, *Listeria monocytogenes*, and *Campylobacter* using multi-year outbreak surveillance data, United States. Atlanta and Washington: US Department of Health and Human Services; 2020.

[3] Painter JA, Hoekstra RM, Ayers T, Tauxe RV, Braden CR, Angulo FJ, et al. Attribution of foodborne illnesses, hospitalizations, and deaths to food commodities by using outbreak data, United States, 1998-2008. Emerg Infect Dis. 2013;19:407–15. 10.3201/eid1903.111866 23622497 PMC3647642

[4] Katz R. State foodborne illness reporting laws, 2011–2013. Inter-university Consortium for Political and Social Research. 2014 [cited 2022 Apr 15]. https://www.icpsr.umich.edu/web/HMCA/studies/34935

[5] Keene BK, Kanwat CP. Enteric disease outbreak investigation and surveillance survey. Presented at: Council for State and Territorial Epidemiologists 2007 Annual Conference; June 24–28, 2007; Atlantic City, NJ, USA.

[6] Centers for Disease Control and Prevention. Epidemiology and Laboratory Capacity for Prevention and Control of Emerging Infectious Diseases (ELC). 2021 [cited 2021 May 19]. https://www.cdc.gov/ncezid/dpei/epidemiology-laboratory-capacity.html

[7] Hedberg C. Guidelines for foodborne disease outbreak response. 3rd edition. Atlanta: Council to Improve Foodborne Outbreak Response; 2020 [cited 2021 May 19]. https://cifor.us/downloads/clearinghouse/CIFOR-Guidelines-Complete-third-Ed.-FINAL.pdf

[8] Centers for Disease Control and Prevention. National *Salmonella* surveillance annual report, 2016. Atlanta: US Department of Health and Human Services; 2018.

[9] Centers for Disease Control and Prevention. National Shiga toxin–producing *Escherichia coli* (STEC) surveillance annual report, 2016. Atlanta: US Department of Health and Human Services; 2018.

[10] Richardson LC, Bazaco MC, Parker CC, Dewey-Mattia D, Golden N, Jones K, et al. An updated scheme for categorizing foods implicated in foodborne disease outbreaks: a tri-agency collaboration. Foodborne Pathog Dis. 2017;14:701–10. 10.1089/fpd.2017.2324 28926300 PMC6317073

[11] Barrett KA, Nakao JH, Taylor EV, Eggers C, Gould LH. Fish-associated foodborne disease outbreaks: United States, 1998–2015. Foodborne Pathog Dis. 2017;14:537–43. 10.1089/fpd.2017.2286 28682115

[12] Dewey-Mattia D, Manikonda K, Hall AJ, Wise ME, Crowe SJ. Surveillance for foodborne disease outbreaks—United States, 2009–2015. MMWR Surveill Summ. 2018;67:1–11. 10.15585/mmwr.ss6710a1 30048426 PMC6061962

[13] Centers for Disease Control and Prevention. An atlas of *Salmonella* in the United States, 1968–2011: laboratory-based enteric disease surveillance. Atlanta: US Department of Health and Human Services; 2013.

[14] Li J, Shah GH, Hedberg C. Complaint-based surveillance for foodborne illness in the United States: a survey of local health departments. J Food Prot. 2011;74:432–7. 10.4315/0362-028X.JFP-10-353 21375880

[15] Jones TF, Rosenberg L, Kubota K, Ingram LA. Variability among states in investigating foodborne disease outbreaks. Foodborne Pathog Dis. 2013;10:69–73. 10.1089/fpd.2012.1243 23249418

[16] Marsh Z, Shah MP, Wikswo ME, Barclay L, Kisselburgh H, Kambhampati A, et al. Epidemiology of foodborne norovirus outbreaks—United States, 2009–2015. Food Saf (Tokyo). 2018;6:58–66. 10.14252/foodsafetyfscj.2017028 32231948 PMC6989197

[17] Ailes E, Budge P, Shankar M, Collier S, Brinton W, Cronquist A, et al. Economic and health impacts associated with a *Salmonella* Typhimurium drinking water outbreak-Alamosa, CO, 2008. PLoS One. 2013;8:e57439. 10.1371/journal.pone.0057439 23526942 PMC3601119

[18] Thomas MK, Vriezen R, Farber JM, Currie A, Schlech W, Fazil A. Economic cost of a *Listeria monocytogenes* outbreak in Canada, 2008. Foodborne Pathog Dis. 2015;12:966–71. 10.1089/fpd.2015.1965 26583272 PMC4691650

[19] Murphree R, Garman K, Phan Q, Everstine K, Gould LH, Jones TF. Characteristics of foodborne disease outbreak investigations conducted by Foodborne Diseases Active Surveillance Network (FoodNet) sites, 2003-2008. Clin Infect Dis. 2012;54(Suppl 5):S498–503. 10.1093/cid/cis232 22572675

[20] Biggerstaff GK. FoodCORE Team. Improving response to foodborne disease outbreaks in the United States. J Public Health Manag Pract. 2015;21:E18–26. 10.1097/PHH.0000000000000115 24983761 PMC4629497

[21] Ebel ED, Williams MS, Cole D, Travis CC, Klontz KC, Golden NJ, et al. Comparing characteristics of sporadic and outbreak-associated foodborne illnesses, United States, 2004–2011. Emerg Infect Dis. 2016;22:1193–200. 10.3201/eid2207.150833 27314510 PMC4918141

[22] Boulton ML, Rosenberg LD; Centers for Disease Control and Prevention (CDC). Food safety epidemiology capacity in state health departments—United States, 2010. MMWR Morb Mortal Wkly Rep. 2011;60:1701–4. 22189892

[23] Association of Public Health Laboratories. PulseNet on the frontlines of foodborne disease surveillance: national molecular subtyping network for foodborne pathogens. APHL Public Health Laboratory Issues in Brief. April 2013 [cited 2022 Apr 17]. https://www.aphl.org/aboutAPHL/publications/Documents/FS_2013April29_PulseNet-on-the-Front-Lines-of-Foodborne-Disease-Surveillance.pdf

[24] Scharff RL, Besser J, Sharp DJ, Jones TF, Peter GS, Hedberg CW. An economic evaluation of PulseNet: a network for foodborne disease surveillance. Am J Prev Med. 2016;50(Suppl 1):S66–73. 10.1016/j.amepre.2015.09.018 26993535

