# Supplementary material for: Foodborne Illness Outbreaks Reported to National Surveillance, United States, 2009–2018
Source: Emerg Infect Dis. 2022 Jun;28(6):1117–27. doi: 10.3201/eid2806.211555 (PMC9155876; doi:10.3201/eid2806.211555)
Supplement: Appendix — Additional information about foodborne illness outbreaks reported to national surveillance, United States, 2009–2018. [file 21-1555-Techapp-s1.pdf]

Article DOI: https://doi.org/10.3201/eid2806.211555

Foodborne Illness Outbreaks Reported to National Surveillance, United States, 2009–2018

Appendix

Appendix Table 1. Structural characteristics of norovirus outbreaks from high- and low-reporting US states by outbreaks per 10 million population, Foodborne Disease Outbreak Surveillance System, United States, 2009–2018

Characteristic	Highest 10 reporters 1,237 outbreaks		Middle 31 reporters 1,480 outbreaks		Lowest 10 reporters 81 outbreaks		p value
Setting identified (n,%)	1,208	97.7	1,396	94.3	72	88.9	<0.01‡
Setting*,† (n,%)							<0.01‡
Restaurant	910	75.3	1,021	73.1	43	59.7	
Institution	44	3.6	40	2.9	7	9.7	
Private residence	44	3.6	26	1.9	1	1.4	
Other single setting	59	4.9	68	4.9	5	6.9	
Multiple setting	151	12.5	241	17.3	16	22.2	
Food vehicle confirmed or suspected (n,%)	318	25.7	361	24.4	14	17.3	0.21‡
Food (n,%)							0.92‡
Multiple	191	60.1	210	58.2	8	57.1	
Aquatic animals	28	8.8	32	8.9	1	7.1	
Land animals	17	5.4	14	3.9	1	7.1	
Plant	62	19.5	78	21.6	0	0.0	
Other§	20	6.3	27	7.5	27	7.5	
Food vehicle confirmed (n,%)	240	75.5	261	72.3	8	57.1	0.24‡
Season (n,%)							<0.01‡
Winter	464	37.5	512	34.6	23	28.4	
Spring	375	30.3	493	33.3	35	43.2	
Summer	147	11.9	235	15.9	12	14.8	
Autumn	251	20.3	240	16.2	11	13.6	
Sex of cases unknown (n,%)	196	8.1	443	8.7	79	12.7	<0.01‡
Number of cases¶ (median, IQR)	10	15	11	17	22	29	<0.01#
Reporting structure							0.69††
Centralized (n,%)	3	30.0	13	41.9	3	30.0	
Decentralized (n,%)	7	70.0	18	58.1	7	10.0	
ELC funding per capita (median, IQR)‡	$0.96	$0.84–1.58	$0.79	$0.44–1.50	$0.45	$0.34–0.89	<0.01#
CDC ELC-funded Foodborne programs**							0.05††
CoE (n,%)	3	30.0	3	9.7	0	0.0	
FoodCORE (n,%)	3	30.0	2	6.5	0	0.0	
OBNE (n,%)	5	50.0	17	54.8	3	30.0	
None (n,%)	1	10.0	9	29.0	5	50.0	
NoroSTAT ‡‡ (n,%)	4	40.0	8	25.8	0	0.0	0.10††
FoodNet §§ (n,%)	4	40.0	6	19.4	0	0.0	0.80††

* Among outbreaks with characteristic identified.
†Restaurant setting includes caterer, banquet hall; Institution includes day cares, hospitals, long-term care facilities/nursing homes/assisted living facilities, prison/jails, and school/college/universities; Other setting category includes camp, fair, festival, other temp or mobile services, farm/dairy, grocery store, hotel/motel, office/indoor workplace, other, religious facility, ship/boat.
‡χ^2 test, comparison across three reporting tiers.
§"Other" foods includes foods which were unclassifiable or invalid using food categories defined by the Interagency Food Safety Analytics Collaboration (8).
¶Laboratory-confirmed and probable primary cases.
#Kruskal-Wallis Test.
** ELC- funding funded foodborne programs: Integrated Food Safety Centers of Excellence (CoE), Foodborne Diseases Centers for Outbreak Response Enhancement (FoodCORE), OutbreakNet Enhanced (OBNE); States with multiple programs were categorized into the category with more funding (e.g., states with CoE and FoodCORE were counted only in CoE) such that program categories are mutually exclusive.
†† Fisher exact test.
‡‡ Norovirus Sentinel Testing and Tracking (NoroSTAT).
§§ Foodborne Diseases Active Surveillance Network.

Appendix Table 2. Structural characteristics of *Salmonella* outbreaks from high- and low-reporting US states by outbreaks per 10 million population, Foodborne Disease Outbreak Surveillance System, United States, 2009–2018

Characteristic	Highest 10 reporters 283 outbreaks		Middle 31 reporters 720 outbreaks		Lowest 10 reporters 188 outbreaks		p value
	No.	%	No.	%	No.	%	
Setting identified (n,%)	235	83.0	650	90.3	142	75.5	<0.01‡
Setting*,† (n,%)							0.01‡
Restaurant	113	48.1	286	44.0	71	50.0	
Institution	8	3.4	33	5.1	14	9.9	
Private residence	38	16.2	85	13.1	25	17.6	
Other single setting	24	10.2	72	11.1	8	5.6	
Multiple setting	52	22.1	174	26.8	24	16.9	
Food vehicle confirmed or suspected (n,%)	114	40.3	292	40.6	71	37.8	0.78‡
Food (n,%)							0.26‡
Multiple	33	29.0	81	27.7	22	31.0	
Aquatic animals	2	1.8	7	2.4	5	7.0	
Land animals	58	50.9	162	55.5	30	42.3	
Plant	18	15.8	35	12.0	10	14.1	
Other§	3	2.6	7	2.4	4	5.6	
Food vehicle confirmed (n,%)	89	78.1	214	73.3	51	71.8	0.54‡
Season (n,%)							0.42‡
Winter	41	14.5	84	11.7	23	12.2	
Spring	75	26.5	171	23.8	51	27.1	
Summer	113	39.9	288	40.0	67	35.6	
Autumn	54	19.1	177	24.6	47	25.0	
Sex of cases unknown (n,%)	14	5.0	39	5.4	6	3.2	0.46‡
Number of cases¶ (median, IQR)	7	11	9	15	7	12	<0.01#
Reporting structure							0.16††
Centralized (n,%)	4	40.0	13	43.3	1	10.0	
Decentralized (n,%)	6	60.0	17	56.7	9	90.0	
ELC funding per capita (median, IQR)‡	$1.16	$0.84–1.58	$0.85	$0.44–1.50	$0.44	$0.33–0.71	0.02#
CDC ELC-funded Foodborne Programs**							0.38††
CoE (n,%)	2	20.0	2	6.7	2	20.0	
FoodCORE (n,%)	2	20.0	3	10.0	0	0.0	
OBNE (n,%)	6	60.0	14	46.7	5	50.0	
None (n,%)	0	0.0	11	36.7	3	30.0	
NoroSTAT ‡‡ (n,%)	4	40.0	7	23.3	1	10.0	0.29††
FoodNet §§ (n,%)	4	40.0	5	16.7	1	10.0	0.19††
Average *Salmonella* incidence rate reported to LEDS¶¶ (mean, standard deviation)	14.5	4.0	16.0	6.0	15.9	10.3	0.86#

*Among outbreaks with characteristic identified.
†Restaurant setting includes caterer, banquet hall; Institution includes day cares, hospitals, long term care facilities/nursing homes/assisted living facilities, prison/jails, and school/college/universities; Other setting category includes camp, fair, festival, other temp or mobile services, farm/dairy, grocery store, hotel/motel, office/indoor workplace, other, religious facility, ship/boat.
‡ χ^2 test, comparison across three reporting tiers.
§"Other" foods includes foods which were unclassifiable or invalid using food categories defined by the Interagency Food Safety Analytics Collaboration (*8*).
¶Laboratory-confirmed and probable primary cases.
#Kruskal-Wallis test.
**ELC- funding funded foodborne programs: Integrated Food Safety Centers of Excellence (CoE), Foodborne Diseases Centers for Outbreak Response Enhancement (FoodCORE), OutbreakNet Enhanced (OBNE); States with multiple programs were categorized into the category with more funding (e.g., states with CoE and FoodCORE were counted only in CoE) such that program categories are mutually exclusive.
††Fisher exact test.
‡‡Norovirus Sentinel Testing and Tracking (NoroSTAT).
§§Foodborne Diseases Active Surveillance Network.
¶¶Average incidence rates per 100,000 population by group reported by states to CDC's Laboratory Enteric Disease System (LEDS), 2016 (6,7).

Appendix Table 3. Structural characteristics of bacterial-toxin outbreaks from high- and low-reporting US states by outbreaks per 10 million population, Foodborne Disease Outbreak Surveillance System, United States, 2009–2018

	Highest 10 reporters 219 outbreaks		Middle 31 reporters 377 outbreaks		Lowest 10 reporters 21 outbreaks		
Characteristic	No.	%	No.	%	No.	%	p-value
Setting identified (n,%)	213	97.3	365	96.8	18	85.7	0.02‡
Setting*,† (n,%)							<0.01‡
Restaurant	141	66.2	206	56.4	3	16.7	
Institution	15	7.0	23	6.3	2	11.1	
Private residence	20	9.4	24	6.6	2	11.1	
Other single setting	7	3.3	28	7.7	3	16.7	
Multiple setting	30	14.1	84	23.0	8	44.4	
Food vehicle confirmed or suspected (n,%)	124	56.6	260	69.0	13	61.9	0.01‡
Food (n,%)							0.45‡
Multiple	50	40.3	121	46.5	7	53.9	
Aquatic animals	1	0.8	3	1.2	0	0.0	
Land animals	62	50.0	100	38.5	4	30.8	
Plant	11	8.9	31	11.9	2	15.4	
Other§	0	0.0	5	1.9	0	0.0	
Food vehicle confirmed (n,%)	82	66.1	204	78.5	12	92.3	0.01‡
Season (n,%)							0.41‡
Winter	39	17.8	81	21.5	6	28.6	
Spring	68	31.1	118	31.3	4	19.1	
Summer	60	27.4	88	23.3	3	14.3	
Autumn	52	23.7	90	23.9	8	38.1	
Sex of cases unknown (n,%)	45	20.6	39	10.3	7	33.3	0.02‡
Number of cases¶ (median, IQR)	8	17	12	25	38	34	<0.01#
Reporting structure							0.26††
Centralized (n,%)	5	50.0	6	23.1	4	40.0	
Decentralized (n,%)	5	50.0	20	76.9	6	60.0	
ELC funding per capita (median, IQR)‡	$1.16	$1.01–1.88	$0.66	$0.44–0.91	$0.44	$0.33–0.67	<0.01#
CDC ELC-funded Foodborne Programs**							<0.01††
CoE (n,%)	2	20.0	4	15.4	0	0.0	
FoodCORE (n,%)	3	30.0	2	7.7	0	0.0	
OBNE (n,%)	4	40.0	15	57.7	5	50.0	
None (n,%)	1	10.0	5	19.2	5	50.0	
NoroSTAT ‡‡ (n,%)	3	30.0	8	30.8	1	10.0	0.42††
FoodNet §§ (n,%)	2	20.0	8	30.8	0	0.0	0.13††

*Among outbreaks with characteristic identified.
†Restaurant setting includes caterer, banquet hall; Institution includes day cares, hospitals, long term care facilities/nursing homes/assisted living facilities, prison/jails, and school/college/universities; Other setting category includes camp, fair, festival, other temp or mobile services, farm/dairy, grocery store, hotel/motel, office/indoor workplace, other, religious facility, ship/boat.
‡χ^2 test, comparison across three reporting tiers.
§"Other" foods includes foods which were unclassifiable or invalid using food categories defined by the Interagency Food Safety Analytics Collaboration (*8*).
¶Laboratory-confirmed and probable primary cases.
#Kruskal-Wallis test.
**ELC- funding funded foodborne programs: Integrated Food Safety Centers of Excellence (CoE), Foodborne Diseases Centers for Outbreak Response Enhancement (FoodCORE), OutbreakNet Enhanced (OBNE); States with multiple programs were categorized into the category with more funding (e.g., states with CoE and FoodCORE were counted only in CoE) such that program categories are mutually exclusive.
††Fisher exact test.
‡‡Norovirus Sentinel Testing and Tracking (NoroSTAT).
§§Foodborne Diseases Active Surveillance Network.

Appendix Table 4. Structural characteristics of STEC O157 outbreaks from high- and low-reporting US states by outbreaks per 10 million population, Foodborne Disease Outbreak Surveillance System, United States, 2009–2018

Characteristic	Highest 10 reporters 59 outbreaks		Middle 31 reporters 61 outbreaks		Lowest 10 reporters 30 outbreaks		p value
	No.	%	No.	%	No.	%	
Setting identified (n,%)	55	9332	53	86.9	24	80.0	0.18‡
Setting*,† (n,%)							0.37‡
Restaurant	23	41.8	13	24.5	10	41.7	
Institution	2	3.6	3	5.7	3	12.5	
Private residence	12	21.8	17	32.1	5	20.8	
Other single setting	4	7.3	7	13.2	3	12.5	
Multiple setting	14	25.5	13	24.5	3	12.5	
Food vehicle confirmed or suspected (n,%)	33	55.9	39	63.9	16	53.3	0.54‡
Food (n,%)							0.22‡
Multiple	4	12.1	6	15.4	1	6.3	
Aquatic animals	0	0.0	1	2.6	0	0.0	
Land animals	20	60.6	26	66.7	7	43.8	
Plant	9	27.3	6	15.4	8	50.0	
Other§	-	-	-	-	-	-	
Food vehicle confirmed (n,%)	22	66.7	31	79.5	12	75.0	0.46‡
Season (n,%)							0.42‡
Winter	9	15.3	8	13.1	4	13.3	
Spring	13	22.0	8	13.1	10	33.3	
Summer	24	40.7	27	44.3	11	36.7	
Autumn	13	22.0	18	29.5	5	16.7	
Sex of cases unknown (n,%)	59	100.0	61	100.0	27	90.0	<0.01‡
Number of cases¶ (median, IQR)	4	6	5	6	7	13	0.19#
Reporting structure							0.31††
Centralized (n,%)	4	40.0	4	28.6	1	10.0	
Decentralized (n,%)	6	60.0	10	71.4	9	90.0	
ELC funding per capita (median, IQR)‡	$1.35	$0.91–2.12	$0.80	$0.59–0.91	$0.43	$0.30–.45	<0.01#
CDC ELC-funded Foodborne Programs**							0.56††
CoE (n,%)	2	20.0	3	21.4	1	10.0	
FoodCORE (n,%)	1	10.0	2	14.3	2	20.0	
OBNE (n,%)	3	30.0	5	35.7	7	70.0	
None (n,%)	4	40.0	4	28.6	0	0.0	
NoroSTAT ‡‡ (n,%)	3	30.0	5	35.7	1	10.0	0.35††
FoodNet §§ (n,%)	2	20.0	6	42.9	2	20.0	0.35††
Average Shiga toxin–producing Escherichia coli incidence rate reported to LEDS¶¶	4.9	3.1	2.4	1.5	2.6	3.3	0.04#

*Among outbreaks with characteristic identified.
†Restaurant setting includes caterer, banquet hall; Institution includes day cares, hospitals, long term care facilities/nursing homes/assisted living facilities, prison/jails, and school/college/universities; Other setting category includes camp, fair, festival, other temp or mobile services, farm/dairy, grocery store, hotel/motel, office/indoor workplace, other, religious facility, ship/boat..
‡χ^2 test, comparison across three reporting tiers.
§Other" foods includes foods which were unclassifiable or invalid using food categories defined by the Interagency Food Safety Analytics Collaboration (8).
¶Laboratory-confirmed and probable primary cases.
#Kruskal-Wallis test.
**ELC- funding funded foodborne programs: Integrated Food Safety Centers of Excellence (CoE), Foodborne Diseases Centers for Outbreak Response Enhancement (FoodCORE), OutbreakNet Enhanced (OBNE); States with multiple programs were categorized into the category with more funding (e.g., states with CoE and FoodCORE were counted only in CoE) such that program categories are mutually exclusive.
††Fisher exact test.
‡‡Norovirus Sentinel Testing and Tracking (NoroSTAT).
§§Foodborne Diseases Active Surveillance Network.
¶¶Average incidence rates per 100,000 population by group reported by states to CDC's Laboratory Enteric Disease System (LEDS), 2016 (6,7).
